# The association between isometric strength and cognitive function in adults with cerebral palsy

**DOI:** 10.3389/fmed.2023.1080022

**Published:** 2023-04-26

**Authors:** Patricia C. Heyn, Alex Tagawa, Zhaoxing Pan, Timothy Reistetter, Ted Kheng Siang Ng, Meredith Lewis, James J. Carollo

**Affiliations:** ^1^Center for Optimal Aging (COA), Marymount University, Arlington, VA, United States; ^2^Physical Medicine & Rehabilitation Department, University of Colorado Anschutz Medical Campus, Aurora, CO, United States; ^3^Center for Gait and Movement Analysis (CGMA), Children’s Hospital Colorado, Aurora, CO, United States; ^4^Musculoskeletal Research Center (MRC), Orthopedics Institute, Children’s Hospital Colorado, Aurora, CO, United States; ^5^University of Texas Health Science Center San Antonio, San Antonio, TX, United States; ^6^Edson College of Nursing and Health Innovation, Arizona State University, Phoenix, AZ, United States

**Keywords:** cerebral palsy - diagnosis, cognitive function, isometric strength measurement, rate of force development (RFD), hand grip dynamometer, aging, frailty, gross motor function classification scale (GMFCS)

## Abstract

**Background:**

The literature supports quantifying the maximum force/tension generated by one’s forearm muscles such as the hand grip strength (HGS) to screen for physical and cognitive frailty in older adults. Thus, we postulate that individuals with cerebral palsy (CP), who are at higher risk for premature aging, could benefit from tools that objectively measure muscle strength as a functional biomarker to detect frailty and cognitive decline. This study assesses the clinical relevancy of the former and quantifies isometric muscle strength to determine its association with cognitive function in adults with CP.

**Methods:**

Ambulatory adults with CP were identified from a patient registry and were enrolled into this study. Peak rate of force development (RFD) and maximum voluntary isometric contraction of the quadriceps were measured using a commercial isokinetic machine, while HGS was collected with a clinical dynamometer. Dominant and non-dominant side were identified. Standardized cognitive assessments, including the Wechsler Memory and Adult Intelligence Scales IV, Short Test of Mental Status, and the Patient-Reported Outcomes Measurement Information System (PROMIS^®^) were used to evaluate cognitive function.

**Results:**

A total of 57 participants (32 females; mean age 24.3 [SD 5.3]; GMFCS levels I–IV) were included in the analysis. Although dominant and non-dominant RFD and HGS measures were associated with cognitive function, non-dominant peak RFD showed the strongest associations with cognitive function.

**Conclusion:**

RFD capacity may reflect age-related neural and physical health and could be a better health indicator than HGS in the CP population.

## 1. Introduction

Cerebral Palsy (CP) is characterized by damage or injury to the developing brain sustained before, during, or shortly after birth, and affects roughly 3.5 individuals per 1,000 live births (1). This makes CP the most common physical disability in children (1). Although CP is considered a childhood condition, adults with CP experience chronic, lifetime disability and often develop a variety of secondary health conditions that could be a sign of premature aging (2). Premature aging is characterized by the development of common geriatric health conditions such as cardiovascular disease, hypertension, cognitive decline, and frailty syndrome at an earlier age (2–5).

Frailty syndrome is a common geriatric condition associated with disease severity and mortality in the older adult population (6, 7). In the absence of a standardized clinical definition, frailty syndrome has been operationalized as cumulative deficits in key musculoskeletal systems such as weak grip strength, low energy, slow walking speed, challenges in performing physical activities, and/or unintentional weight loss (6, 7). Individuals with CP often have difficulty performing physical activities including routine daily tasks due to musculoskeletal impairments, sarcopenia, and pain (1, 3, 5). This may contribute to experiencing signs of frailty much earlier compared to adults without any pathology as is often reported (5–7). Therefore, there is a need for research evaluating innovative screening, diagnostics, biomarkers, and interventions to delay disease development, decrease frailty, and the subsequent risk for early co-morbidities and mortality in adults with CP (2–5).

Hand grip strength (HGS) is recognized as an important health indicator and is commonly used to measure upper body neuromusculoskeletal function (8–10). Substantial evidence supports that HGS is a strong predictor of disability and frailty in the older adult population (11). HGS is also strongly correlated with cognitive function in the older population (12). Additionally, HGS has been associated with function and activities of daily living in children and adults with CP (13). While HGS is a good measurement tool to evaluate neuromuscular function in the upper extremities, it might not be the most sensitive measure to capture physical frailty and functional health decline in adults with CP. HGS is a simple and unidimensional measure of maximum strength and does not assess the speed of muscle contraction (14–16). This limits the assessment of neurofunctional abilities for individuals with CP (13), as many individuals with CP have hypertonicity, including spasticity and dystonia (1). The physical metric known as Rate of Force Development (RFD) has the potential to be a more multidimensional measure of neuromuscular health for populations with musculoskeletal impairments because it includes a measure of both the speed and magnitude of a muscle group’s force output (14–16). RFD measures the rate at which force, or torque, is produced during a specific amount of time and is dependent on muscle strength, type of muscle fibers, muscle size, and motor neuron function (17). Moreau and colleagues (14, 15) reported the importance of RFD when assessing fatigue and functional ability in individuals with CP and concluded that RFD may be of greater importance than maximal strength for specific tasks. They also stated that interventions focused on improving RFD would also improve the functional ability of individuals with CP (14, 15). Similarly, other studies have reported that high RFD values are needed to counteract the effects of sudden changes in balance (17), which pertains to adults with CP as they have reduced overall balance and are at an elevated risk for falling (18, 19).

While RFD is a promising neuromuscular health assessment tool for adults with CP, there is limited literature reporting the associations between RFD and important neurologic and functional outcomes associated with frailty in adults with CP. Specifically, whether RFD could be a good correlate of physical and cognitive outcomes in adults with CP is unknown; as such, this study aimed to evaluate the associations between RFD, HGS, and cognitive function in adults with CP to shed light on the potential integration of these instruments as a potential health screening tool to capture early physical and cognitive frailty in adults with CP before they develop severe frailty syndrome and disability (20).

## 2. Materials and methods

### 2.1. Setting and study design

This report analyzed data collected from the parent study; “The Cerebral Palsy Adult Transition Study” (CPAT) which has been previously described (2, 3, 21, 22). The CPAT study was performed at a clinical motion analysis laboratory in a regional children’s hospital that has been serving the health needs of individuals with CP for over 20 years (2, 3, 21, 22). The facility is accredited by the Commission for Motion Laboratory Accreditation (CMLA^1^) and is composed of a multidisciplinary team of clinicians and researchers. The study was approved by the university’s Institutional Review Board and all participants signed informed consent prior to participation.

### 2.2. Participants

Participants with a confirmed diagnosis of CP over the age of 18 years were identified from an internal patient registry and were invited to participate in a short telephone screening survey to determine if they were (1) interested and able to participate in the study, and (2) able to walk across a 35-foot (10.6 m) walkway, with or without assistive devices, at least three times. A total of 72 ambulatory participants passed the study inclusion criteria and participated in the parent CPAT study (2, 3, 21, 22). For this study evaluating the associations between peak RFD of the quadriceps, HGS, and cognitive function in ambulatory adults with CP, data from 57 participants out of the 72 were used for the analysis. A total of 15 participants did not complete the cognitive and physical performance assessments and were not included in the analysis (Table 1).

**Table 1:** Demographics.

Age	
Mean Age (SD) [range]	24.3 (5.3) [18–48]
Gender (*n*)	
Male	25
Female	32
Ethnicity (n)	
African-American	3
Asian/Pacific Islander	1
Hispanic/Latino	7
White	39
Mixed/Multiethnic	6
Other	1
CP Diagnosis (n)	
Right hemiplegia	9
Left hemiplegia	13
Diplegia	30
Triplegia	3
Quadriplegia	2
Education Level	
Mean Years (SD)	13.8 (2.4)
GMFCS (*n*)	
I	25
II	18
III	13
IV	1

### 2.3. Assessment tools and outcomes

#### 2.3.1. Isometric knee extension strength assessment

Calculation of isometric strength outcome variables was performed in compliance with guidelines published by Moreau and colleagues (14). Testing was performed during an isometric knee extension activity using the HUMAC NORM isokinetic dynamometer (CSMi, Stoughton, MA). Participants were seated and stabilized at an 85° back angle with a fixed 60° knee flexion angle. Each subject was instructed to push as hard as they could for 5 s followed by 60 s of rest between exercise trials. Three exercise trials were collected. The maximum voluntary isometric contraction (MVIC) of each trial was identified from the knee extension torque vs. time curve, and the slope of the rising edge (rate of force development, RFD) was calculated at 0–30, 0–50, 0–100, and 0–200 ms, where *t0* was defined as the time when muscle torque either met or first exceeded 2.5% of the maximum torque value. The peak RFD corresponds to the single highest slope value between 0–30, 0–50, 0–100, and 0–200 ms intervals. Additionally, RFD50 was calculated as the slope at 50% of MVIC. Successful trials were initially identified as having a starting torque of 0. If an offset was present, in which the trial had a non-zero resting torque, the measured offset was subtracted from the MVIC to adjust for this residual force, and the procedure described above was performed. Trials were excluded from analysis if they did not follow the Moreau et al. protocol (14) or if the trial did not have a definite offset. If multiple trials for a given leg were successful, the trial with the largest Peak RFD was used in the analysis. These tests were performed on both limbs, in which the right and the left side were analyzed separately (14–16).

#### 2.3.2. Cognitive function assessments

Cognitive function was measured using the Wechsler Memory Scale-IV (WMS-IV) (23, 24), the Wechsler Adult Intelligence Scale-IV (WAIS-IV) (23, 24), the Short Test of Mental Status (STMS) (25), and the Patient-Reported Outcomes Measurement Information System (PROMIS®) Applied Cognition–Executive Function (26, 27). PROMIS® is a large measurement information system initiative funded by the National Institutes of Health that has been extensively evaluated, validated, and used in reported outcomes research (26). The following WMS-IV subtests were used to evaluate a participant’s memory (23): the Visual Reproduction I, Logical Memory I, Verbal Paired Association I, Category Fluency Test, Visual Reproduction II Delayed Recall, Visual Reproduction II Recognition, Logical Memory II Delayed Recall, Logical memory II, Visual Reproduction II Delayed Recall, Visual Reproduction II, and Visual Reproduction II Word Recall. The WAIS-IV subtests were used to evaluate a person’s overall cognitive ability, which consisted of Block Design, Digit Span, Symbol Search, and Picture Completion (24). The STMS was used to evaluate the global cognition and the overall cognitive status of the study participants (25). Qualified and trained research staff administered the neuropsychological protocol.

#### 2.3.3. Hand grip strength

Bilateral hand grip strength (HGS) was obtained by a trained research staff member using a Jamar digital hand dynamometer (model number 5030 J1, Patterson Medical, Warrenville, IL, United States). This model of dynamometer was chosen due to its proven value in CP patient populations (28). After adjusting for hand size, participants were instructed and verbally encouraged to grip the handle of the dynamometer as hard as possible with their dominant and non-dominant hands; participants performed this test while seated, shoulders adducted and neutrally rotated, elbow flexed at 90, with forearms in a neutral position and wrist between 0 and 30 degrees of dorsiflexion. Three trials were collected and averaged for analysis. This procedure for HGS data collection follows the recommendations of the The American Society of Hand Therapists (29).

#### 2.3.4. Dominant side identification

For individuals who were diagnosed with hemiplegia, a form of unilateral CP, the non-dominant side was identified as the affected side. For those who had a diagnosis of diplegia, triplegia, or quadriplegia, forms of bilateral CP, dominant and non-dominant sides were identified by asking participants which hand they wrote with, in which their writing hand was considered their dominant side (20). For those who were not able to write and unable to communicate which side was their dominant side, the values from their left and right side were averaged, in which the average value was used as both their dominant and non-dominant values.

### 2.4. Statistical analysis

With a power of 80% to detect statistical significance at a 5% alpha level, the study was powered to detect significant correlations (≥ 0.33). Mean plus standard deviation and percentage distribution summarized continuous and categorical outcomes, respectively. Isokinetic values were calculated for both the dominant and non-dominant side using the methods published by Moreau et al. (16). The predictor variables (Peak RFD, MVIC, RFD50, and HGS) were correlated with the WMS-IV raw scores, WAIS-IV raw scores, and STMS total scores to identify associations using Pearson’s correlation coefficient. After the bivariate analysis, a comparison between dominant and non-dominant Peak RFD, MVIC, RFD50, and HGS was performed to identify which variable showed the strongest correlations (determined by the largest r2 value) and had the greatest number of correlations with all cognitive outcome variables. Subsequently, a multi-variable linear regression was performed, in which the outcome variables used were the cognitive functional assessment sub-tests that had the strongest correlations with the isometric knee extension strength assessment and the hand grip strength variables. A backward selection method was used to determine which predictor variables significantly impacted the final model, in which non-significant variables were taken out of the model, one predictor at a time, until only the statistically significant variables were left in the model. The predictor variables included in the full model were dominant and non-dominant MVIC, Peak RFD, RFD50, and HGS. In step 1 of the analyses, due to the exploratory nature of the bivariate analyses, no statistical adjustments were performed. In step 2 of the analyses, for the multi-linear regression, significance was adjusted using the Bonferroni correction, in which a value of p under 0.00625 (0.05/8) was considered statistically significant. SAS 9.4 (SAS Institute Inc., Cary, NC, United States) was used as the program for all analyses.

## 3. Results

A total of 57 participants were included in this study. The demographics of this cohort comprised 25 males and 32 females, who had a mean age of 24.3 [SD 5.3] with a GMFCS level between I–IV. Full demographic information is provided in Table 1.

### 3.1. Association between isometric assessment and cognitive function

**Figure 1:**
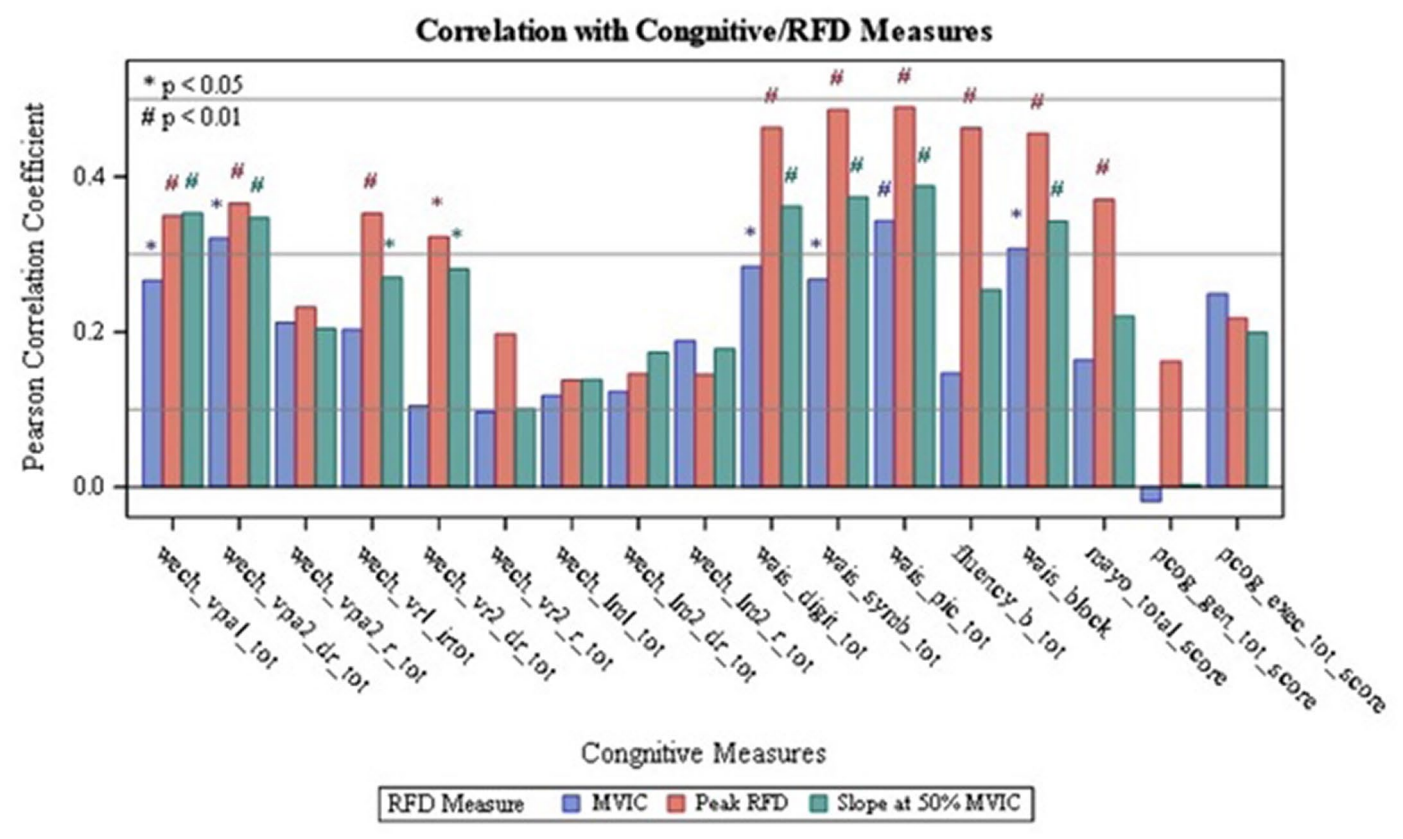
Correlation of Dominant RFD with Cognitive Measures: wech_vpal_tot — Total Visual Paired Associates I — Immediate Recall Raw Score; wech_vpa2_dr_tot — Total Verbal Paired Associates II-Delayed Recall Raw Score; wech_vpa2_r_tot — Total Verbal Paired Associates II- Recognition Raw Score: wech_vrl_irtot — Total Visual Reproduction I - Immediate Recall Raw Score; wech_vr2_dr_tot — Total Visual Reproduction II — Delayed Recall Raw Score; wech_vr2_r_tot — Total Visual Reproduction II - Recognition Raw Score; wech_Im1_tot — Total Logical Memory I- Immediate Recall Raw Score; wech_lm2_dr_tot — Total Logical Memory II-Delayed Recall Raw Score; wech_Im2_r_tot — Total Logical Memory II-Recognition Raw Score; wais_digit_tot — Total Digit Span Raw Score (forward+backward+sequencing); wais_symb_tot — Total Symbol Search Raw Score, wais_pic_tot — Total Picture Completion Raw Score; fluency_b_tot — Category Test Raw Score; wais_block — Total Block Design Raw Score; mayo_total_score — STMS Total Score; pcog_gen_tot_score — PROMIS Applied Cognition - General Concerns Short Form 8a; pcog_exec_tot_score — Neuro-QOL Applied Cognition-Executive Function.

**Figure 2:**
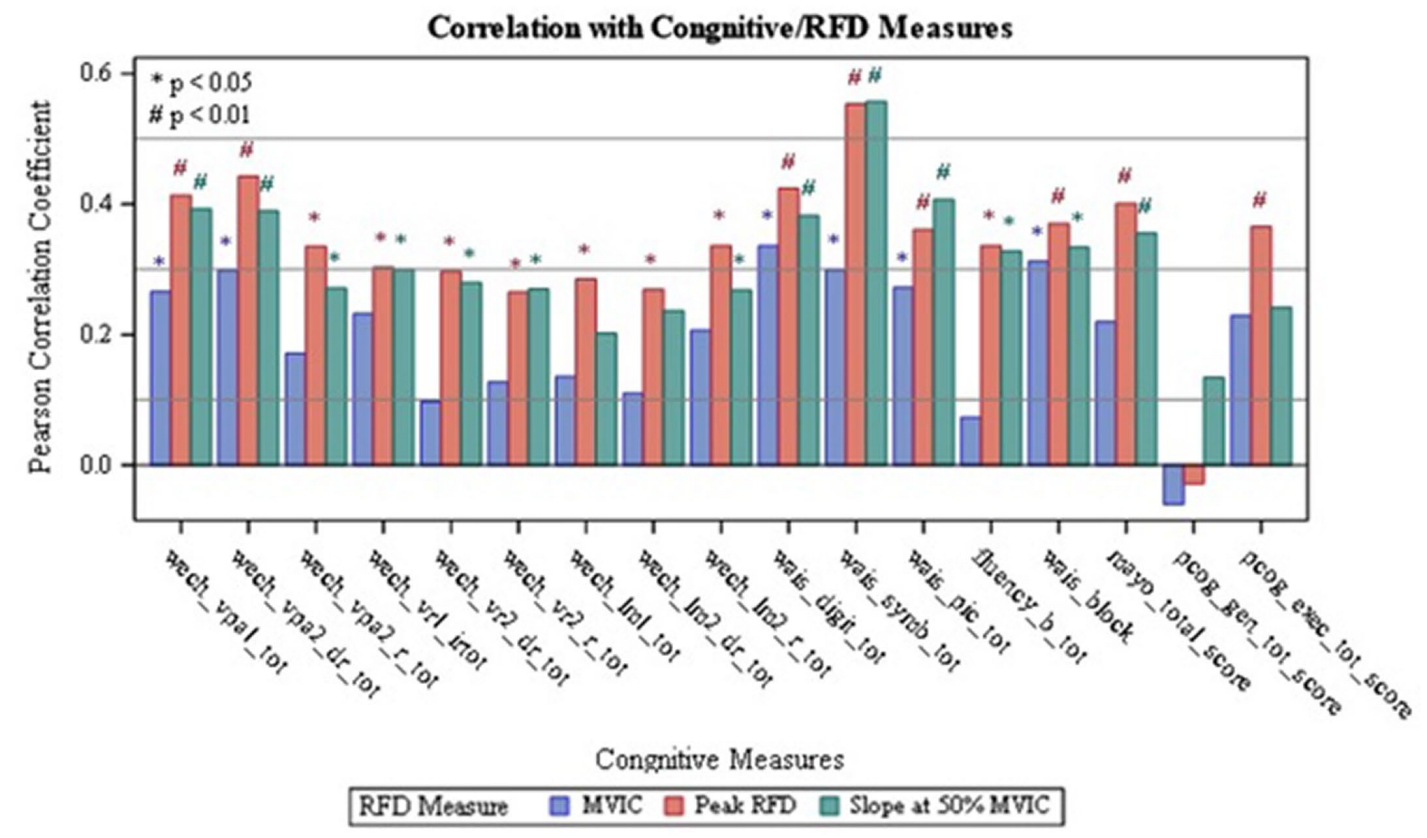
Correlation of Non-dominant RFD with Cognitive Measures; wech_vpal_tot — Total Visual Paired Associates I — Immediate Recall Raw Score; wech_vpa2_dr_tot — Total Verbal Paired Associates II — Delayed Recall Raw Score; wech_vpa2_r_tot — Total Verbal Paired Associates II- Recognition Raw Score; wech_vrl_irtot — Total Visual Reproduction I - Immediate Recall Raw Score; wech_vr2_dr_tot — Total Visual Reproduction II - Delayed Recall Raw Score; wech_vr2_r_tot — Total Visual Reproduction II — Recognition Raw Score: wech_Im1_tot — Total Logical Memory I — Immediate Recall Raw Score; wech_lm2_dr_tot — Total Logical Memory II — Delayed Recall Raw Score, wech_Im2_r_tot — Total Logical Memory II- Recognition Raw Score; wais_digit_tot — Total Digit Span Raw Score (forward+backward+sequencing), wais_symb_tot — Total Symbol Search Raw Score; wais_pic_tot — Total Picture Completion Raw Score; fluency_b tot Category Test Raw Score; wais_block_Total Block Design Raw Score; mayo_total_score — STMS Total Score; pcog_gen_tot_score — PROMIS Applied Cognition - General Concerns Short Form 8a; pcog_exec_tot_score — Neuro-QOL Applied Cognition - Executive Function.

Results on the correlations between the dominant and non-dominant MVIC, Peak RFD, and RFD50 with tests of cognitive function are summarized in Table 2 and Figures 1, 2. Additionally, Supplementary Tables S1 and S2 report the overall results of the analysis. The analysis revealed that all isometric knee extension strength assessment variables had at least one statistically significant correlation with one of the cognitive tests (*p* < 0.05). Upon analyzing the dominant side, it was found that Peak RFD on the dominant side had the greatest number of correlations with the cognitive tests compared MVIC and RFD50 on the dominant side (Table 2). Additionally, Peak RFD had the strongest correlation compared to MVIC and RFD50 on the dominant side, which was indicated by the largest Pearson’s R value. This correlation was between Peak RFD and the Symbol Search sub-test (Pearson’s R value = 0.49) within the WAIS-IV (Table 2, Figure 1, and Supplementary Table S1). Furthermore, it was found that Peak RFD on the dominant side showed strong correlations (*p* < 0.001 and Pearson’s R > 0.40) with all the cognitive sub-tests within the WAIS-IV (Figure 1 and Supplementary Table S1).

**Table 2:** Overall summary of all correlation based on sidedness.

Sidedness	RFD/HGS	Number of statistically significant correlations Pearson (out of 17)	Range of statistically significant correlations Pearson	Cognitive tests correlations		Cognitive function Domain
				Smallest Pearson’s *R*	Largest Pearson’s *R*	
*Dominant*	MVIC	6	0.27–0.34	Visual Paired Associates I - Immediate Recall Raw Score	Picture Completion Raw Score	Visual Perception/Perceptual Organization
	Peak RFD	10	0.32–0.49	Visual Reproduction II-Recognition Raw Score	Symbol Search Raw Score	Processing Speed
	RFD at 50% MVIC	8	0.27–0.39	Visual Reproduction I - Immediate Recall Raw Score	Picture Completion Raw Score	Visual Perception/Perceptual Organization
	HGS	9	0.30–0.45	Total Logical Memory I - Immediate Recall Raw Score	STMS Total Score	Global Cognition
*Non-Dominant*	MVIC	7	0.23–0.34	Visual Reproduction I - Immediate Recall Raw Score	Digit Span Raw Score	Working Memory
	Peak RFD	16	0.26–0.55	Visual Reproduction II - Recognition Raw Score	Symbol Search Raw Score	Processing Speed
	RFD at 50% MVIC	13	0.27–0.56	Verbal Paired Associates II - Recognition Raw Score	Symbol Search Raw Score	Processing Speed
	HGS	9	0.25–0.46	Symbol Search Raw Score	Digit Span Raw Score	Working Memory

RFD, Peak Rate of force development; MVIC, Maximum Voluntary Isometric Contraction; HGS, Hand Grip Strength.

This summarizes the dominant and non-dominant MVIC, Peak RFD, RFD at 50% MVIC, and HGS univariate correlations with cognitive function, in which specific cognitive tests with the smallest and largest Pearson’s R value are displayed.

Upon analyzing the non-dominant side, it was found that Peak RFD on the non-dominant side had the greatest number of correlations with the cognitive tests compared to MVIC and RFD50 on the non-dominant side (Table 2). Additionally, the analysis revealed that the strongest correlations, which were indicated by the largest Pearson’s R value, were observed from Peak RFD and RFD50 variables. These correlations were with the Symbol Search sub-test (Pearson’s R value with Peak RFD = 0.55; Pearson’s R value with RFD50 = 0.56) within the WAIS-IV (Table 2, Figure 2, and Supplementary Table S2). Furthermore, while Peak RFD had the greatest number of correlations with the cognitive test on the non-dominant side, the strong correlations on the non-dominant side (*p* < 0.001 and Pearson’s R > 0.40) were not associated with a single cognitive test (Figure 2 and Supplementary Table S2).

### 3.2. Association between HGS and cognitive function

**Figure 3:**
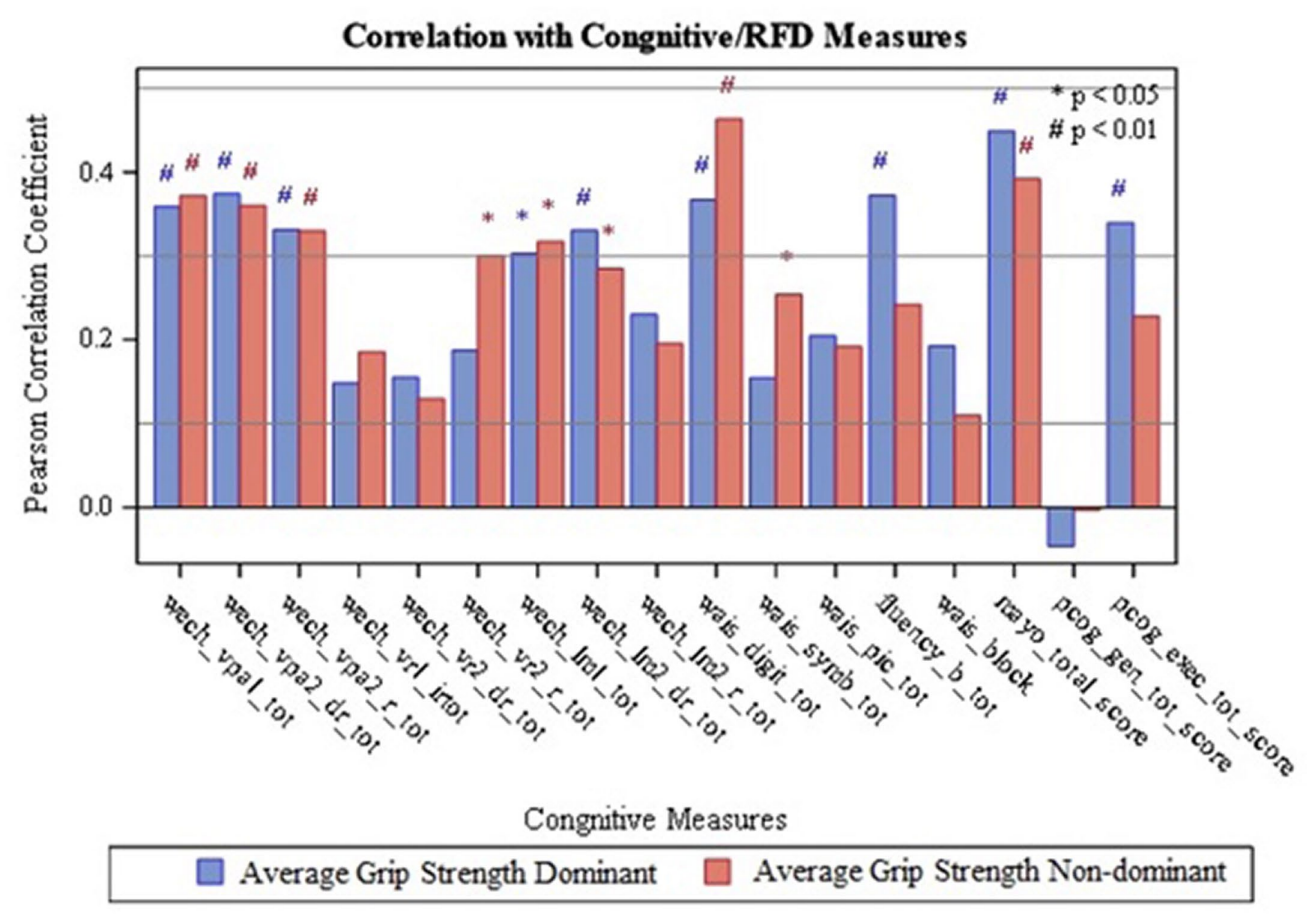
Correlation of Grip strength with Cognitive Measures; wech_vpal_tot — Total Visual Paired Associates I — Immediate Recall Raw Score; wech_vpa2_dr_tot — Total Verbal Paired Associates II-Delayed Recall Raw Score; wech_vpa2_r_tot — Total Verbal Paired Associates II- Recognition Raw Score; wech_vrl_irtot — Total Visual Reproduction I — Immediate Recall Raw Score; wech_vr2_dr_tot — Total Visual Reproduction II - Delayed Recall Raw Score; wech_vr2_r_tot — Total Visual Reproduction II — Recognition Raw Score; wech_Im1_tot — Total Logical Memory I- Immediate Recall Raw Score; wech 1m2_dr_tot — Total Logical Memory II — Delayed Recall Raw Score; wech_Im2_r_tot — Total Logical Memory II — Recognition Raw Score; wais_digit_tot — Total Digit Span Raw Score (forward+backward+sequencing); wais_symb_tot — Total Symbol Search Raw Score, wais_pic_tot - Total Picture Completion Raw Score; fluency_b_tot — Category Test Raw Score; wais_block — Total Block Design Raw Score; mayo_total_score — STMS Total Score: pcog_gen_tot_score — PROMIS Applied Cognition - General Concerns Short Form 8a; pcog exec_tot_score — Neuro-QOL Applied Cognition-Executive Function.

Results analyzing HGS on the dominant and non-dominant side are summarized and presented in Table 2, Figure 3, and Supplementary Table S3. It was observed that dominant and non-dominant HGS had the same number of correlations (total of 9 correlations) with cognitive function (Table 2 and Supplementary Table S3). It was also found that the strongest correlations observed were between non-dominant grip strength and the Digit Span sub-test (Pearson’s R value = 0.46; value of *p*<0.05) (Figure 3 and Supplementary Table S3). Furthermore, the strong correlations for the HGS on the dominant and non-dominant side (*p* < 0.001 and Pearson’s R > 0.40) were not associated with a single cognitive assessment test (Figure 3 and Supplementary Table S3).

### 3.3. Comparison and multi-variable linear regression analysis

Comparison between dominant and non-dominant MVIC, Peak RFD, RFD50, and HGS with the cognitive assessment variables can be summarized in Table 2. This comparison revealed that Peak RFD on the non-dominant side had the second strongest correlations and the highest number of correlations with all the cognitive function tests (WMS/ STM, WAIS-IV and PROMIS®) compared to HGS, MVIC, and RFD50 on either the dominant or non-dominant hand.

Since the Digit Span subtest in the WAIS-IV showed the strongest correlation with the HGS assessment variables (Pearson’s R value = 0.46; value of *p*<0.05), and since the Symbol Search sub-test within the WAIS-IV showed the strongest correlations with the isometric strength assessment variables, these cognitive sub-tests were used as our outcome variables for the multi-variable linear regression analysis. For the model with Digit Span as the outcome variable, the backward selection method excluded every predictor variable except the non-dominant Peak RFD. This resulted in the following final model: Digit Span Score = 19.10052 + 0.03252 x [Non-dominant Peak RFD] (model value of *p* = 0.0006; *r*^2^ = 0.204). Similarly, for the model with Symbol Search as the outcome variable, the backward selection method excluded every predictor variable except the non-dominant Peak RFD. This resulted in the following final model: Symbol Search Score = 13.81751 + 0.06363 x [Non-dominant Peak RFD] (model value of *p* <0.0001; *r*^2^ = 0.3055).

## 4. Discussion

The main findings of this study reveal that higher isometric strength, defined by MVIC, Peak RFD, RFD50, and HGS, was associated with higher cognitive function scores, especially in the executive function domain. Executive function is a set of mental skills that include working memory, attention, speed, flexible thinking, and self-control, which are skills essential for learning, working, and managing daily life. More specifically, Peak RFD and RFD50 showed the strongest association with the Symbol Search Test, which could indicate that higher isometric strength is associated with better processing speed ability such as mental speed defined by the time it takes a person to do a mental task. In other words, processing speed is the time between receiving and responding to a stimulus. These findings support current literature, as multiple studies have found that measures of strength or tasks that are associated with strength and speed have been correlated with cognitive function in other populations (30–32). However, there is limited literature on cognitive function and HGS for individuals with CP. In this population, there have been multiple references showing that HGS and strength are associated with participation and quality of life (33, 34). Additionally, HGS and hand impairments are not directly related to functional ability but do indirectly affect functional skills, as it is one of many contributing factors that impact daily activities and functional ability (13, 34, 35). Current literature has also analyzed the impact of increasing upper and lower body strength in children and adults with CP, in which it is recommended that both children and adults with CP include physical activity in their lives, as it has immense health benefits (36–38). While strength measures are important and have been extensively reported in the CP population, there is no evidence supporting the association between cognitive function and HGS in adults with CP. Many studies focus on whether strength and cognition impact certain areas, such as quality of life and mortality, but very few studies focus on relating HGS and cognition (39, 40). As such, this study is original and innovative in extending the knowledge of current literature by focusing on grip strength, RFD, and cognitive function outcomes in ambulatory adults with CP (see Table 3)

**Table 3:** Overall summary of Mean (SD) data for subgroups.

Variable name	*N*	Mean ± SD	Mean(SD), Females (*N* = 32)	Mean(SD), MALES (*N* = 25)	Mean(SD), BILATERAL CP (*N* = 35)	Mean(SD), Unilateral CP (*N* = 22)
Age	57	24.3 ± 5.3	24.31 ± 6.67	24.20 ± 2.96	24.11 ± 4.82	24.50 ± 6.17
Years of education (highest year of school completed)	57	13.8 ± 2.4	13.66 ± 2.22	13.88 ± 2.57	13.63 ± 2.07	13.95 ± 2.80
Weight (kg)	57	64.2 ± 15.7	60.59 ± 11.93	68.81 ± 18.75	64.44 ± 16.14	63.80 ± 15.33
Height (in)	57	162.8 ± 11.5	156.36 ± 8.49	171.09 ± 9.55	161.75 ± 12.33	164.53 ± 10.23
Body Mass Index (BMI)	57	24.2 ± 4.9	24.87 ± 5.13	23.23 ± 4.42	24.66 ± 5.29	23.34 ± 4.08
RFD 0–30 ms slope Non-Dominant Leg	57	101.8 ± 78.5	92.87 ± 73.94	113.22 ± 84.13	96.36 ± 71.45	110.43 ± 89.68
RFD 0–50 ms slope Non-Dominant Leg	57	107.3 ± 82.4	96.29 ± 75.47	121.29 ± 90.13	105.44 ± 79.12	110.16 ± 89.21
RFD 0–100 ms Slope Non-Dominant Leg	57	108.0 ± 83.7	97.63 ± 80.12	121.30 ± 87.81	108.31 ± 82.97	107.54 ± 86.69
RFD 0–200 slope Non-Dominant Leg	57	107.4 ± 81.8	94.11 ± 73.17	124.47 ± 90.26	107.49 ± 76.59	107.32 ± 91.29
RFD 0–100 ms slope Dominant Leg	53	152.0 ± 119.3	137.04 ± 112.26	171.45 ± 127.79	121.34 ± 104.61	198.65 ± 127.50
RFD 0–200 slope Dominant Leg	53	137.3 ± 93.6	120.01 ± 86.88	159.78 ± 99.01	117.80 ± 90.62	166.94 ± 92.20
RFD 0–30 ms slope Dominant Leg	53	149.6 ± 135.3	132.89 ± 121.00	171.34 ± 151.97	110.85 ± 101.85	208.60 ± 159.57
RFD 0–50 ms slope Dominant Leg	53	156.8 ± 133.0	138.60 ± 118.30	180.56 ± 149.43	120.48 ± 108.60	212.18 ± 149.64
Total Visual Paired Associates I – Immediate Recall Raw Score	57	29.9 ± 15.5	30.88 ± 14.00	28.56 ± 17.48	31.23 ± 15.35	27.68 ± 15.89
Total Verbal Paired Associates II – Delayed Recall Raw Score	55	9.1 ± 4.2	9.16 ± 3.84	9.13 ± 4.77	9.52 ± 4.39	8.59 ± 4.01
Total Verbal Paired Associates II – Recognition Raw Score	55	36.4 ± 6.2	37.35 ± 4.54	35.17 ± 7.73	36.97 ± 4.95	35.55 ± 7.71
Total Visual Reproduction I – Immediate Recall Raw Score	57	29.5 ± 10.2	33.00 ± 7.61	25.08 ± 11.46	29.23 ± 9.99	30.00 ± 10.75
Total Visual Reproduction II – Delayed Recall Raw Score	57	22.5 ± 11.7	26.56 ± 10.03	17.28 ± 11.80	21.34 ± 11.27	24.32 ± 12.40
Total Visual Reproduction II – Recognition Raw Score	57	5.2 ± 1.8	5.69 ± 1.45	4.48 ± 2.00	5.14 ± 1.85	5.18 ± 1.76
Total Logical Memory I – Immediate Recall Raw Score	57	26.2 ± 9.7	27.41 ± 8.12	24.56 ± 11.45	27.63 ± 9.38	23.82 ± 10.03
Total Logical Memory II – Delayed Recall Raw Score	57	22.8 ± 10.2	24.03 ± 8.15	21.12 ± 12.25	23.86 ± 9.64	21.00 ± 10.93
Total Logical Memory II – Recognition Raw Score	56	22.7 ± 3.8	23.44 ± 3.37	21.79 ± 4.15	23.06 ± 3.01	22.23 ± 4.76
Total Digit Span Raw Score (forward+backward+sequencing)	56	23.1 ± 7.2	23.13 ± 5.15	23.08 ± 9.36	23.91 ± 6.46	21.86 ± 8.17
Total Number Correct	55	24.0 ± 10.3	26.03 ± 10.28	21.26 ± 9.88	22.59 ± 10.41	26.38 ± 9.91
Total Symbol Search Raw Score	55	22.6 ± 10.8	24.97 ± 10.40	19.35 ± 10.68	20.94 ± 11.03	25.33 ± 10.06
Total Picture Completion Raw Score	57	9.9 ± 4.0	10.44 ± 3.77	9.32 ± 4.31	9.60 ± 4.05	10.50 ± 3.99
Overall Total words F + S + A	55	32.0 ± 16.0	31.78 ± 14.07	32.26 ± 18.62	32.06 ± 12.88	31.86 ± 20.36
Total Block Design Raw Score	56	27.8 ± 15.5	31.06 ± 13.98	23.33 ± 16.60	23.94 ± 14.89	34.10 ± 14.71
STMS total score	57	30.8 ± 5.0	31.80 ± 3.66	29.44 ± 6.21	31.11 ± 4.77	30.20 ± 5.50
Total Score of PROMIS Applied Cognition	57	16.8 ± 7.4	15.31 ± 6.82	18.64 ± 7.78	16.54 ± 5.73	17.14 ± 9.58
Total score of executive function of Neuro-QOL Applied Cognition	57	48.8 ± 11.5	50.72 ± 10.92	46.28 ± 12.05	48.17 ± 11.52	49.73 ± 11.78

Another major finding from this study is related to the non-dominant Peak RFD. This study revealed that compared to dominant Peak RFD, MVIC, RFD50, and HGS, non-dominant Peak RFD had the second strongest correlations and the highest number of correlations with all the cognitive function tests (WMS/STM, WAIS-IV, and PROMIS®). Furthermore, upon performing the multi-variable linear regression using the backward selection method, dominant Peak RFD, non-dominant HGS, MVIC, and RFD50 were taken out of the model, revealing that Peak RFD on the non-dominant side was the only statistically significant predictor variable that correlated with the cognitive assessment variables. These results suggest that Peak RFD on the non-dominant side is a better predictor of cognitive function compared to all other strength assessment variables, as it is the strongest predictor variable correlated with cognitive function, and Peak RFD is correlated with a broad spectrum of cognitive domains. Similar findings have been reported in the literature, as Moreau et al. (14) also found that Peak RFD on the non-dominant side correlated with functional ability in individuals with CP. An explanation as to why these results were observed could be due to a hierarchical relationship among performance measures used to characterize physical and cognitive function. From the General Systems Performance Theory model of human performance developed by Kondraske (41, 42), higher-level physical and cognitive tasks, such as walking and information processing speed, respectively, require sufficient amounts of more basic elements of human performance, such as strength or speed, to achieve a specified level of performance in the higher-level task. The theory further suggests that higher-level task performance may be limited by any one of the more basic performance “resources” such as strength, balance, and coordination, suggesting that it is the weaker or less dominant side that more greatly influences the overall task performance (41, 42). When applying these theories to the study findings, we believe Peak RFD shows a stronger correlation with cognitive function outcomes compared to the HGS because it reflects the subject’s ability to accomplish two fundamental human tasks, force production and rate (speed) of force production simultaneously, whereas HGS only reflects force production ability without regard to how quickly the force can be applied or removed. For individuals with CP, who have greater challenges with fine motor control and characteristically may have greater difficulty with complex and high-level tasks, overall cognitive performance may be better characterized by RFD than HGS, and subsequently may be a more robust screening tool than the unidimensional assessment provided by HGS test.

Regardless, our results reveal that compared to HGS, non-dominant Peak RFD has a stronger correlation with cognitive function, especially with cognitive tests that required mental speed and attention. This may indicate that non-dominant RFD has the potential as a biomarker or index for cognitive decline. In the literature, adults with CP are showing signs of accelerated aging (2, 43) such as developing secondary health conditions earlier in life (2–5). We also previously showed that several neurocognitive functions were at comparable levels between the two groups (2). To identify these changes earlier, we would need precise and accurate indicators to help monitor or measure changes in performance as individuals with CP age. While HGS is unidimensional as a screening tool (8, 9, 11, 12), peak RFD’s ability to describe both the magnitude and speed of force development may be a more robust and comprehensive tool, and better suited for screening for cognitive decline and secondary health conditions; especially so in individuals with CP, which uniquely affects physical and muscular health, necessitating more complex assessments/instruments (20). RFD is unique in that it is a quantitative measure that also directly describes an individual’s functional ability and task performance (18, 20).

Previous studies support the cognitive and motor function relationship in individuals with cerebral palsy (44, 45). Rooijen et al. (44) showed that the cognitive and motor predictors were positively correlated with each other in a sample of children with CP. Their study showed that word decoding task and fine motor skills were the strongest predictors of arithmetic performance among children with CP. This pioneering work by Rooijen’s group combined with the results of our study will help future research to evaluate the relationships between measures of physical strength and cognitive function in other populations. Specifically, an idea for future research into RFD and cognitive function amongst the pediatric and pre-teenager CP populations could give valuable insight into the growth and development of individuals with CP as they transition to adult healthcare. Thus, the authors would be interested in furthering this research with other tests of cognitive function, physical performance, and quality of life (44, 45).

An earlier study by our research team (43) supported the association between mobility performance and participation and executive function in adults with CP. Therefore, integrating screening and measures that capture early changes in functional performance, may assist practitioners in the identification of key functional deficits associated with diseases as well as premature cognitive decline in adults with CP. Thus, healthcare practitioners should adopt screenings that can identify early markers associated with physical and cognitive frailty to intervene and prescribe personalized interventions that will increase and include individuals with CP in physical activities. Their participation in these interventions could improve their cognitive and motor function and could assist and increase the quality of life of individuals with CP.

By screening for changes in performance that may be linked to deficits in function and premature cognitive aging in adults with CP, clinicians can develop targeted interventions to increase participation in physical activities, which may simultaneously improve cognitive outcomes and more fully engage individuals with CP in their communities. This group would be interested in exploring further investigations into relationships between measures of physical strength and cognition in other populations. Specifically, an idea for future research into RFD and cognition amongst the pediatric and teen CP populations could give valuable insight into the growth and development of individuals with CP. Previous studies have shown the relationship between word development, fine motor skill development, and arithmetic performance in children with CP; the authors would be interested in furthering this research with other tests of cognitive function and physical performance (44).

## 5. Limitations

A limitation of the current investigation is that while the parent CPAT study is relatively large for a longitudinal functional outcome study of adults with CP, the cross-sectional adult cohort represents a sample that is relatively small and diverse for determining prevalence across the CP population. Due to the cross-sectional study nature, we could not conclude the directions of causality for the associations detected. Larger sample size with multiple time points would address this limitation and could possibly reveal differences in key markers associated with premature disease and aging. Another limitation of this study is the generalizability of the study. Since the parent study focused on gait performance and secondary disease development in individuals who are ambulatory, individuals at GMFCS levels IV and V were underrepresented. As such, further validation of these findings would require similar investigation in individuals with CP who rely primarily on wheeled mobility. Another limitation would be how the dominant side is defined. Since dominance was determined by asking participants with bilateral CP, there could be errors in which dominance was identified. To address this issue, a more rigorous definition of defining the dominant side should be used in future studies. Lastly, owing to the pilot and exploratory nature of this study, we did not control for multiple testing. These considerations suggest that while noteworthy, this work is a starting point for additional research evaluating RFD in a broader sample of adults with CP.

## 6. Conclusion

This study found an association between RFD, HGS, and cognitive function in ambulatory adults with CP. Upon comparison between RFD and HGS, Peak RFD was correlated with a higher number of cognitive function measures and had a stronger correlation with common cognitive tests than the HGS. Because RFD is a higher-level functional task than HGS and provides information about both magnitude and rate of force production, it could be used as a promising screening tool for measuring both functional and cognitive decline in this vulnerable population.

## Data availability statement

The original contributions presented in the study are included in the article/Supplementary material, further inquiries can be directed to the corresponding author.

## Ethics statement

The studies involving human participants were reviewed and approved by Colorado Multiple Institutional Review Board. The patients/participants provided their written informed consent to participate in this study.

## Author contributions

PH, TR, ZP, and JC: conceptualization methodology. ZP: software and formal Analysis. PH, AT, ZP, TR, and JC: validation, investigation, writing – Review & Editing, and visualization. PH and AT: data Curation and writing – original draft preparation. PH and JC: supervision, project administration, and funding acquisition. All authors contributed to the article and approved the submitted version.

## Funding

This research was supported by grants from the National Institute on Disability, Independent Living, and Rehabilitation Research (NIDILRR #H133G130200, NIDILRR #90IF0055-01), in the Administration for Community Living (ACL) of the Department of Health and Human Services (HHS). Additional support was provided from the J. T. Tai & Company Foundation.

## Conflict of interest

The authors declare that the research was conducted in the absence of any commercial or financial relationships that could be construed as a potential conflict of interest.

## Publisher’s note

All claims expressed in this article are solely those of the authors and do not necessarily represent those of their affiliated organizations, or those of the publisher, the editors and the reviewers. Any product that may be evaluated in this article, or claim that may be made by its manufacturer, is not guaranteed or endorsed by the publisher.

## Footnotes

^1^ www.CMLAinc.org

## Abbreviations

CP: Cerebral Palsy
HGS: Hand grip strength
RFD: Rate of Force Development
MVIC: Maximum Voluntary Isometric Contraction
CPAT Study: Cerebral Palsy Adult Transition Study
WMS-IV: Wechsler Memory Scale IV
WAIS-IV: Wechsler Adult Intelligence Scale IV
PROMIS^®^: Patient-Reported Outcomes Measurement Information System
STMS: Short Test of Mental Status
