# Supplementary material for: The association between isometric strength and cognitive function in adults with cerebral palsy
Source: Front Med (Lausanne). 2023 Apr 26;10:1080022. doi: 10.3389/fmed.2023.1080022 (PMC10170265; doi:10.3389/fmed.2023.1080022)
Supplement: Supplementary file 1 [file Table_1.docx]

Supplementary Material

Supplementary Table 1: Dominant Side RFD Associations with Cognitive Outcomes

| **RFD Variables for Dominant Side** | **Cognitive Outcome** | **Pearson r** | **p for Pearson r** | **p<0.05** |
| --- | --- | --- | --- | --- |
| Maximum Voluntary Isometric Contraction (MVIC) | Total Visual Paired Associates I - Immediate Recall Raw Score | 0.27 | 0.045 | Y |
|  | Total Verbal Paired Associates II - Delayed Recall Raw Score | 0.32 | 0.017 | Y |
|  | Total Verbal Paired Associates II - Recognition Raw Score | 0.21 | 0.120 |  |
|  | Total Visual Reproduction I - Immediate Recall Raw Score | 0.20 | 0.130 |  |
|  | Total Visual Reproduction II - Delayed Recall Raw Score | 0.10 | 0.440 |  |
|  | Total Visual Reproduction II - Recognition Raw Score | 0.10 | 0.475 |  |
|  | Total Logical Memory I - Immediate Recall Raw Score | 0.12 | 0.381 |  |
|  | Total Logical Memory II - Delayed Recall Raw Score | 0.12 | 0.362 |  |
|  | Total Logical Memory II - Recognition Raw Score | 0.19 | 0.168 |  |
|  | Total Digit Span Raw Score (forward+backward+sequencing) | 0.28 | 0.032 | Y |
|  | Total Symbol Search Raw Score | 0.27 | 0.044 | Y |
|  | Total Picture Completion Raw Score | 0.34 | 0.009 | Y |
|  | Category Test Raw Score | 0.15 | 0.285 |  |
|  | Total Block Design Raw Score | 0.31 | 0.021 | Y |
|  | STMS Total Score | 0.16 | 0.222 |  |
|  | PROMIS Applied Cognition – General Concerns Short Form 8a | -0.02 | 0.892 |  |
|  | Neuro-QOL Applied Cognition – Executive Function | 0.25 | 0.062 |  |
| Peak RFD | Total Visual Paired Associates I - Immediate Recall Raw Score | 0.35 | 0.008 | Y |
|  | Total Verbal Paired Associates II - Delayed Recall Raw Score | 0.37 | 0.006 | Y |
|  | Total Verbal Paired Associates II - Recognition Raw Score | 0.23 | 0.089 |  |
|  | Total Visual Reproduction I - Immediate Recall Raw Score | 0.35 | 0.007 | Y |
|  | Total Visual Reproduction II - Delayed Recall Raw Score | 0.32 | 0.014 | Y |
|  | Total Visual Reproduction II - Recognition Raw Score | 0.20 | 0.141 |  |
|  | Total Logical Memory I - Immediate Recall Raw Score | 0.14 | 0.308 |  |
|  | Total Logical Memory II - Delayed Recall Raw Score | 0.15 | 0.278 |  |
|  | Total Logical Memory II - Recognition Raw Score | 0.15 | 0.291 |  |
|  | Total Digit Span Raw Score (forward+backward+sequencing) | 0.46 | <.001 | Y |
|  | Total Symbol Search Raw Score | 0.49 | <.001 | Y |
|  | Total Picture Completion Raw Score | 0.49 | <.001 | Y |
|  | Category Test Raw Score | 0.46 | <.001 | Y |
|  | Total Block Design Raw Score | 0.46 | <.001 | Y |
|  | STMS Total Score | 0.37 | 0.005 | Y |
|  | PROMIS Applied Cognition – General Concerns Short Form 8a | 0.16 | 0.228 |  |
|  | Neuro-QOL Applied Cognition – Executive Function | 0.22 | 0.104 |  |
| Slope at 50% MVIC | Total Visual Paired Associates I - Immediate Recall Raw Score | 0.35 | 0.007 | Y |
|  | Total Verbal Paired Associates II - Delayed Recall Raw Score | 0.35 | 0.009 | Y |
|  | Total Verbal Paired Associates II - Recognition Raw Score | 0.20 | 0.134 |  |
|  | Total Visual Reproduction I - Immediate Recall Raw Score | 0.27 | 0.042 | Y |
|  | Total Visual Reproduction II - Delayed Recall Raw Score | 0.28 | 0.034 | Y |
|  | Total Visual Reproduction II - Recognition Raw Score | 0.10 | 0.457 |  |
|  | Total Logical Memory I - Immediate Recall Raw Score | 0.14 | 0.304 |  |
|  | Total Logical Memory II - Delayed Recall Raw Score | 0.17 | 0.197 |  |
|  | Total Logical Memory II - Recognition Raw Score | 0.18 | 0.194 |  |
|  | Total Digit Span Raw Score (forward+backward+sequencing) | 0.36 | 0.006 | Y |
|  | Total Symbol Search Raw Score | 0.37 | 0.004 | Y |
|  | Total Picture Completion Raw Score | 0.39 | 0.003 | Y |
|  | Category Test Raw Score | 0.25 | 0.061 |  |
|  | Total Block Design Raw Score | 0.34 | 0.010 | Y |
|  | STMS Total Score | 0.22 | 0.100 |  |
|  | PROMIS Applied Cognition – General Concerns Short Form 8a | 0.00 | 0.983 |  |
|  | Neuro-QOL Applied Cognition – Executive Function | 0.20 | 0.137 |  |

Supplementary Table 2: Non-Dominant Side RFD Associations with Cognitive Outcomes

| **RFD Variables for Non- Dominant Side** | **Cognitive Outcome** | **Pearson r** | **p for Pearson r** | **p<0.05** |
| --- | --- | --- | --- | --- |
| Maximum Voluntary Isometric Contraction (MVIC) | Total Visual Paired Associates I - Immediate Recall Raw Score | 0.27 | 0.046 | Y |
|  | Total Verbal Paired Associates II - Delayed Recall Raw Score | 0.30 | 0.027 | Y |
|  | Total Verbal Paired Associates II - Recognition Raw Score | 0.17 | 0.211 |  |
|  | Total Visual Reproduction I - Immediate Recall Raw Score | 0.23 | 0.083 | Y |
|  | Total Visual Reproduction II - Delayed Recall Raw Score | 0.10 | 0.474 |  |
|  | Total Visual Reproduction II - Recognition Raw Score | 0.13 | 0.345 |  |
|  | Total Logical Memory I - Immediate Recall Raw Score | 0.14 | 0.313 |  |
|  | Total Logical Memory II - Delayed Recall Raw Score | 0.11 | 0.415 |  |
|  | Total Logical Memory II - Recognition Raw Score | 0.21 | 0.127 |  |
|  | Total Digit Span Raw Score (forward+backward+sequencing) | 0.34 | 0.011 | Y |
|  | Total Symbol Search Raw Score | 0.30 | 0.027 | Y |
|  | Total Picture Completion Raw Score | 0.27 | 0.041 | Y |
|  | Category Test Raw Score | 0.07 | 0.595 |  |
|  | Total Block Design Raw Score | 0.31 | 0.019 | Y |
|  | STMS Total Score | 0.22 | 0.101 |  |
|  | PROMIS Applied Cognition – General Concerns Short Form 8a | -0.06 | 0.658 |  |
|  | Neuro-QOL Applied Cognition – Executive Function | 0.23 | 0.087 |  |
| Peak RFD slope | Total Visual Paired Associates I - Immediate Recall Raw Score | 0.41 | 0.001 | Y |
|  | Total Verbal Paired Associates II - Delayed Recall Raw Score | 0.44 | <.001 | Y |
|  | Total Verbal Paired Associates II - Recognition Raw Score | 0.34 | 0.012 | Y |
|  | Total Visual Reproduction I - Immediate Recall Raw Score | 0.30 | 0.022 | Y |
|  | Total Visual Reproduction II - Delayed Recall Raw Score | 0.30 | 0.025 | Y |
|  | Total Visual Reproduction II - Recognition Raw Score | 0.26 | 0.047 | Y |
|  | Total Logical Memory I - Immediate Recall Raw Score | 0.29 | 0.032 | Y |
|  | Total Logical Memory II - Delayed Recall Raw Score | 0.27 | 0.043 | Y |
|  | Total Logical Memory II - Recognition Raw Score | 0.34 | 0.011 | Y |
|  | Total Digit Span Raw Score (forward+backward+sequencing) | 0.42 | 0.001 | Y |
|  | Total Symbol Search Raw Score | 0.55 | <.001 | Y |
|  | Total Picture Completion Raw Score | 0.36 | 0.006 | Y |
|  | Category Test Raw Score | 0.34 | 0.012 | Y |
|  | Total Block Design Raw Score | 0.37 | 0.005 | Y |
|  | STMS Total Score | 0.40 | 0.002 | Y |
|  | PROMIS Applied Cognition – General Concerns Short Form 8a | -0.03 | 0.834 |  |
|  | Neuro-QOL Applied Cognition – Executive Function | 0.37 | 0.005 | Y |
| Slope at 50% MVIC | Total Visual Paired Associates I - Immediate Recall Raw Score | 0.39 | 0.003 | Y |
|  | Total Verbal Paired Associates II - Delayed Recall Raw Score | 0.39 | 0.003 | Y |
|  | Total Verbal Paired Associates II - Recognition Raw Score | 0.27 | 0.045 | Y |
|  | Total Visual Reproduction I - Immediate Recall Raw Score | 0.30 | 0.024 | Y |
|  | Total Visual Reproduction II - Delayed Recall Raw Score | 0.28 | 0.035 | Y |
|  | Total Visual Reproduction II - Recognition Raw Score | 0.27 | 0.042 | Y |
|  | Total Logical Memory I - Immediate Recall Raw Score | 0.20 | 0.131 |  |
|  | Total Logical Memory II - Delayed Recall Raw Score | 0.24 | 0.077 |  |
|  | Total Logical Memory II - Recognition Raw Score | 0.27 | 0.046 | Y |
|  | Total Digit Span Raw Score (forward+backward+sequencing) | 0.38 | 0.004 | Y |
|  | Total Symbol Search Raw Score | 0.56 | <.001 | Y |
|  | Total Picture Completion Raw Score | 0.41 | 0.002 | Y |
|  | Category Test Raw Score | 0.33 | 0.015 | Y |
|  | Total Block Design Raw Score | 0.33 | 0.012 | Y |
|  | STMS Total Score | 0.36 | 0.007 | Y |
|  | PROMIS Applied Cognition – General Concerns Short Form 8a | 0.13 | 0.320 |  |
|  | Neuro-QOL Applied Cognition – Executive Function | 0.24 | 0.070 |  |

Supplementary Table 3: Dominant/Non-Dominant Side Grip Strength with Cognitive Outcomes

| **Grip Strength Variables** | **Cognitive Outcome** | **Pearson r** | **p for Pearson r** | **p<0.05** |
| --- | --- | --- | --- | --- |
| Grip Strength for dominant hand | Total Visual Paired Associates I - Immediate Recall Raw Score | 0.36 | 0.003 | Y |
|  | Total Verbal Paired Associates II - Delayed Recall Raw Score | 0.37 | 0.002 | Y |
|  | Total Verbal Paired Associates II - Recognition Raw Score | 0.33 | 0.007 | Y |
|  | Total Visual Reproduction I - Immediate Recall Raw Score | 0.15 | 0.234 |  |
|  | Total Visual Reproduction II - Delayed Recall Raw Score | 0.16 | 0.214 |  |
|  | Total Visual Reproduction II - Recognition Raw Score | 0.19 | 0.132 |  |
|  | Total Logical Memory I - Immediate Recall Raw Score | 0.30 | 0.012 | Y |
|  | Total Logical Memory II - Delayed Recall Raw Score | 0.33 | 0.006 | Y |
|  | Total Logical Memory II - Recognition Raw Score | 0.23 | 0.063 |  |
|  | Total Digit Span Raw Score (forward+backward+sequencing) | 0.37 | 0.002 | Y |
|  | Total Symbol Search Raw Score | 0.15 | 0.219 |  |
|  | Total Picture Completion Raw Score | 0.20 | 0.097 |  |
|  | Category Test Raw Score | 0.37 | 0.002 | Y |
|  | Total Block Design Raw Score | 0.19 | 0.121 |  |
|  | STMS Total Score | 0.45 | <.001 | Y |
|  | PROMIS Applied Cognition – General Concerns Short Form 8a | -0.05 | 0.706 |  |
|  | Neuro-QOL Applied Cognition – Executive Function | 0.34 | 0.004 | Y |
| Grip Strength for non-dominant hand | Total Visual Paired Associates I - Immediate Recall Raw Score | 0.37 | 0.002 | Y |
|  | Total Verbal Paired Associates II - Delayed Recall Raw Score | 0.36 | 0.004 | Y |
|  | Total Verbal Paired Associates II - Recognition Raw Score | 0.33 | 0.009 | Y |
|  | Total Visual Reproduction I - Immediate Recall Raw Score | 0.19 | 0.145 |  |
|  | Total Visual Reproduction II - Delayed Recall Raw Score | 0.13 | 0.312 |  |
|  | Total Visual Reproduction II - Recognition Raw Score | 0.30 | 0.017 | Y |
|  | Total Logical Memory I - Immediate Recall Raw Score | 0.32 | 0.011 | Y |
|  | Total Logical Memory II - Delayed Recall Raw Score | 0.28 | 0.022 | Y |
|  | Total Logical Memory II - Recognition Raw Score | 0.20 | 0.128 |  |
|  | Total Digit Span Raw Score (forward+backward+sequencing) | 0.46 | <.001 | Y |
|  | Total Symbol Search Raw Score | 0.25 | 0.046 | Y |
|  | Total Picture Completion Raw Score | 0.19 | 0.128 |  |
|  | Category Test Raw Score | 0.24 | 0.058 |  |
|  | Total Block Design Raw Score | 0.11 | 0.392 |  |
|  | STMS Total Score | 0.39 | 0.001 | Y |
|  | PROMIS Applied Cognition – General Concerns Short Form 8a | -0.00 | 0.979 |  |
|  | Neuro-QOL Applied Cognition – Executive Function | 0.23 | 0.066 |  |
